# Use of coagulation screening in the critical care unit

**DOI:** 10.1186/cc11035

**Published:** 2012-03-20

**Authors:** A Rice, R Paterson, C Cairns

**Affiliations:** 1 Forth Valley Royal Hospital, Larbert, UK

## Introduction

The aim of this audit is to compare the effectiveness of indiscriminate coagulation testing versus selective testing based on clinical indications within the HDU setting. Coagulation tests (PT and APTT) are often taken as a matter of routine alongside patient's daily blood tests in the critical care setting. Abnormal coagulation results rarely alter patient management while repeated testing has significant detrimental financial implications.

## Methods

Over a 14-day period, the blood results of HDU patients were prospectively analysed in order to assess whether or not a coagulation screen was conducted and whether or not this was appropriate based on clinical indications. Following targeted education towards medical and nursing staff, including publicising a list of clinical indications within the unit, the audit cycle was repeated.

## Results

Prior to education, only 37% of coagulation screens were clinically indicated. Following implementation of the indications this rose to 50%. Using the guidelines in the second round there was 100% identification of abnormal results compared to only 81% prior to education. On review of all these data we were able to extrapolate that prior to targeted education there was a 2:1 ratio of appropriate to inappropriate coagulation testing, post intervention this rose to 5:1.

## Conclusion

With local targeted education of staff we significantly reduced the number of inappropriate coagulation tests undertaken within our unit from 65% to 27%. Along with this we had a 100% detection rate for abnormal results using our list of clinical indications for testing. In our high turnover critical care unit this would indicate potential savings of around £10,000 per annum; a significant amount in an organisation with longstanding financial constraints.

